# Mobility gene expression differences among wild-type, *Mmp20* null and *Mmp20* over-expresser mice plus visualization of 3D mouse ameloblast directional movement

**DOI:** 10.1038/s41598-023-44627-0

**Published:** 2023-11-01

**Authors:** Masashi Shin, Aya Matsushima, Jun-ichi Nagao, Yoshihiko Tanaka, Hidemitsu Harada, Koji Okabe, John D. Bartlett

**Affiliations:** 1 Section of Cellular Physiology, Department of Physiological Science and Molecular Biology, Fukuoka Dental College, 2-15-1 Tamura, Sawara-ku, Fukuoka, 814-0193 Japan; 2 Oral Medicine Research Center, Fukuoka Dental College, Fukuoka, Japan; 3 Section of Infection Biology, Department of Functional Bioscience, Fukuoka Dental College, Fukuoka, Japan; 4 Divison of Developmental Biology and Regenerative Medicine, Department of Anatomy, Iwate Medical University, Iwate, Japan; 5 Division of Biosciences, College of Dentistry, Ohio State University, Columbus, OH USA

**Keywords:** Enamel, Extracellular matrix

## Abstract

Enamel forming ameloblasts move away from the dentino-enamel junction and also move relative to each other to establish enamel shape during the secretory stage of enamel development. Matrix metalloproteinase-20 (MMP20) is a tooth specific proteinase essential for proper enamel formation. We previously reported that MMP20 cleaves cadherins and may regulate ameloblast movement. Here, we used an *Amelx* promoter driven tdTomato reporter to label mouse ameloblasts. With these transgenic mice, we assessed ameloblast mobility group dynamics and gene expression. Three-dimensional imaging of mouse ameloblasts were observed in hemi-mandibles by using a tissue clearing technique. The three-dimensional ameloblast layer in Tg(*Amelx*-*Mmp20*) mice that overexpress MMP20 was uneven and the ameloblasts migrated away from this layer. Mouse ameloblast movement toward incisal tips was monitored by ex vivo time-lapse imaging. Gene expression related to cell migration and adhesion was analyzed in ameloblasts from wild-type mice, *Mmp20*^−/−^ mice with no functional MMP20 and from Tg(*Amelx*-*Mmp20*) overexpressing mice. Gene expression was altered in *Mmp20*^−/−^ and Tg(*Amelx*-*Mmp20*) mice compared to wild type. Among the genes assessed, those encoding laminins and a gap junction protein were upregulated in *Mmp20*^−/−^ mice. New techniques and findings described in this study may lead to an improved understanding of ameloblast movement during enamel formation.

## Introduction

Tooth formation starts with the epithelium and continues via reciprocal epithelial-mesenchymal interactions^1^. Epithelial stem cells give rise to transiently-amplifying cells that may differentiate into ameloblasts responsible for enamel formation^2^. By using live cell imaging, a population of *Fgf8*-expressing epithelial cells, which condensed in dental lamina, were demonstrated to migrate to the proper position so that tooth formation could initiate^3^. It was previously demonstrated that an early epithelial signaling center governs tooth budding morphogenesis^4^. Early tooth development was observed dynamically in these prior results, but the enamel developmental stages that include ameloblast movement were not investigated.

Ameloblasts secrete enamel matrix proteins including amelogenin encoded by *Amelx* and initiate enamel ribbons during the secretory stage and these ribbons grow in width and thickness during the subsequent maturation stage of enamel development. The secretory stage ameloblasts move away from dentino-enamel junction (DEJ) as the enamel ribbons elongate^5,6^. Approximately 10,000 enamel ribbons will grow in width and thickness and will eventually fuse into an enamel rod. Enamel rods show the trail of the migrating ameloblasts as they move away from the DEJ. The forces that control ameloblast movement were proposed in the 1970’s^7^ and ameloblast movement was studied by examining enamel rod patterns with histological analyses^8^, but living ameloblast dynamic movement within an organism has not previously been reported.

Matrix metalloproteinase-20 (MMP20, enamelysin) is a tooth specific proteinase that cleaves enamel matrix proteins during the secretory stage^9^. MMPs hydrolyze extracellular matrix and regulate cell adhesion and migration in development, disease and carcinogenesis^10^. Mutations in *MMP20* cause amelogenesis imperfecta (AI) in humans^11,12^ and *Mmp20* knockout mice also display an AI phenotype^13^. Previously, we showed that MMP20 cleaved cadherins leading to β-catenin translocation to the cell nucleus^14,15^. Overexpression of MMP20 in *Mmp20* Transgenic (Tg) mice caused a disrupted ameloblast layer and the enamel was less well mineralized than normal^16^.

In this study we generated reporter mice to label ameloblasts. These mice allowed us to observe living ameloblast movement dynamics in ex vivo organ culture and to isolate highly purified stage specific mouse ameloblasts. We performed gene expression analyses in these purified cells and found notable cell migration and adhesion related genes that alter gene expression in the absence of *Mmp20* and when *Mmp20* is overexpressed.

## Materials and methods

### Availability of data and software

Gene expression data were deposited in the Gene Expression Omnibus (GEO) at (https://www.ncbi.nlm.nih.gov/geo/). The GEO accession number is GSE233825. Cell migration and adhesion genes (accession number, GO:0016477 and GO:0098609) were selected by Amigo (http://amigo.geneontology.org/amigo) from scRNA-seq data^17^ present in the reference’s supplementary Table S1^17^, top 100 enriched genes in ameloblasts and present in the reference’s supplementary Table S3^17^ with 439 genes in the ameloblast trajectory for a total 539 genes. 53 out of the 539 genes were selected as cell migration and adhesion genes. Note that 26 of the 53 genes were evaluated (Fig. 3, Table 2), 18 genes were not detected by qPCR, and 9 genes were not analyzed.

### Animals

Experimental protocols were approved by the Fukuoka Dental College Experimental Animal Committee (Approval numbers: 17026, 22010) for the humane use of animals. All procedures were performed in accordance with the Guidelines for Proper Conduct of Animal Experiments of the Science Council of Japan and were performed in compliance with ARRIVE guidelines. *Mmp20*^−/−^ and Tg(*Amelx*-*Mmp20*) mice were previously generated^13,16^. In previous reports, Tg(*Amelx*-*Mmp20*) mice are the mice denoted as *Mmp20*^+/+^ Tg or Tg24 (high MMP20 expresser)^15,16^. *Amelx-tdTomato* (AT) mice were generated in this study. The *tdTomato* was excised from *pcDNA3.1*(+)*/Luc2* = *tdT* vector (Addgene, 32904) by restriction digestion with *Bam*HI*-Eco*RI and was then blunt-ended. The *pCR2.1-Amelx promoter-Mmp20* vector^16^ was digested with *Asc*I*-Asi*SI and the *tdTomato* replaced the *Mmp20* cDNA in the blunt-ended *pCR2.1-Amelx promoter* vector. This vector contained 4639 bp of mouse *Amelx* promoter including the first exon (non-coding) and first intron; immediately 3' to the *tdTomato* and 1127 bp of *Amelx* 3' non-coding region as previously constructed^18^. The *Amelx promoter-tdTomato* with the 3'-*Amelx* non-coding region was excised from the vector by restriction digestion with *Not*I*–Spe*I, purified with a Qiaquick gel extraction kit (Qiagen) and microinjected into fertilized oocytes for surgical transfer to recipients. Germline transmission was determined by PCR analyses of genomic DNA obtained from tail biopsies. PCR primers used to identify the presence of the transgene were: forward, 5'-CCTAGCTTCTAGGAATCAGTATTCG-3'; reverse, 5'-ATTTGTAGGAGCAAATGACCAGTCCC-3'. Two transgenic founders were obtained and both lines were maintained as homozygotes. The AT mice were genotyped with tail DNA using PCR analyses with the following primers: forward, 5'-GTGCAACTGCCCGGCTACTACTAC-3'; reverse, 5'-ATTTGTAGGAGCAAATGACCAGTCCC-3'. AT mice were mated with *Mmp20*^−/−^ to obtain *Mmp20*^+/-^-AT mice and then *Mmp20*^+/-^-AT mice were crossed with each other to produce *Mmp20*^−/−^-AT mice. AT mice were mated with Tg(*Amelx*-*Mmp20*) to produce Tg(*Amelx*-*Mmp20*)-AT mice.

### In situ hybridization

An RNAscope 2.5HD Red detection kit (Advanced Cell Diagnostics, 322350) was used according to the manufacturer’s instructions. The tissues were formalin-fixed, decalcified in a solution of 20% w/v sodium citrate/4% v/v formic acid paraffin-embedded, and sectioned. The tissue sections were boiled in the target retrieval solution at 98 °C for 10 min and incubated in Protease Plus solution at 40 °C for 30 min. The sections were incubated with a probe for Mus musculus *Amelx*, 441851 (Advanced Cell Diagnostics) at 40 °C for 2 h. Hematoxylin was used as a counterstain. Images were obtained using a microscope (BZX710, Keyence Corp.).

### Frozen section

AT mouse hemimandibles were dissected, immersed in 4% paraformaldehyde (PFA) fixative overnight, and washed in PBS five times during 5 h. The tissues were immersed in 15% sucrose and 30% sucrose overnight respectively for cryoprotection and embedded in SCEM (Section-Lab). The blocks were cryosectioned by Kawamoto’s film method at 5-μm thickness at −30 °C. The films were rinsed with PBS, counterstained with DAPI, and mounted with ProLong Diamond antifade reagent. The sections were examined by fluorescence microscopy (BZX710, Keyence Corp.).

### FACS sorting

Incisor enamel organ epithelial cells were isolated and enzymatically processed in 4w/v% collagenase (Fujifilm Wako Chemicals) in Dulbecco’s modified Eagle’s medium (DMEM)/Ham’s F12 (Fujifilm Wako Chemicals) for 30 min at room temperature, 0.5w/v% trypsin–EDTA (Fujifilm Wako Chemical) for 10 min at 37 ℃ followed by neutralization with DMEM/Ham’s F12 containing 10% fetal bovine serum (FBS). Single cell suspensions were filtered with a cell strainer (40 μm, Corning), and then subjected to fluorescence-activated cell sorting (FACS) to isolate tdTomato positive and negative cells using FACSMelody (BD Biosciences). Sorted cells were centrifuged 1000 rpm for 5 min and pellets were lysed in RA1 buffer (Macherey–Nagel) for RNA extraction.

### RNA isolation and quantitative PCR (qPCR)

RNA was extracted from ∼6000 sorted cells using a NucleoSpin RNA XS (Macherey–Nagel). Each RNA sample was extracted from an individual mouse. RNA concentration was measured by Agilent 2100 Bioanalyzer (Agilent Technologies). Ten ng RNA was transcribed to cDNA using PrimeScript RT Master Mix (Takara Bio). Reactions were performed on a CFX96 Real-Time System (Bio-Rad) using the following program: 40 cycles at 95 ℃ for 10 s and 55 ℃ for 30 s. Subsequently, a melting curve was prepared. The primers used in this study are listed in Supplementary Table S1. All measurements were normalized to *Gapdh* and the results were determined using the 2^−ΔΔCT^ method.

### Tissue clearing

The hemimandibles from AT mice were dissected and cleared with CUBIC (Clear, Unobstructed Brain/Body Imaging Cocktails and Computational analysis) as previously described^19^. Hemimandibles were fixed with 4% PFA at 4 °C overnight and washed with PBS at room temperature for a day. The samples were immersed in CUBIC-L (TCI chemicals) with shaking at 37 °C for 3 days followed by PBS washing. The samples were then immersed in CUBIC-B (TCI chemicals) with shaking at 37 °C for 5 days followed by PBS washing. The samples were subsequently immersed in CUBIC-L with shaking at 37 °C for 2 days followed by PBS washing. Finally, the samples were immersed in 50% CUBIC-R+ (TCI chemicals) in water with shaking at room temperature for a day and then with CUBIC-R+ with shaking for a day. The images were obtained by Lightsheet Z.1 fluorescence microscope (Carl Zeiss Microscopy). Data processing and 3D rendering was by use of Arivis Vision 4D software (https://www.arivis.com). Imaging parameters were: Zeiss EC Plan-Neofluar 5×/0.16 lens, volume size: 7.3 × 4.4 × 3.4 mm, 450 z-sections, three images were tiled.

### Time-lapse imaging

Incisors were dissected from 4–5 day old postnatal mouse mandibles in Dulbecco’s phosphate buffered saline (PBS). The extracted incisors were stained with 5 μg/ml Hoechst33342 solution (Dojindo) and cultured in DMEM/Ham’s F12 containing 10% FBS, 100 units/ml penicillin, and 100 mg/ml streptomycin (Meiji Seika Pharma Co.) on glass bottom dishes (D11130H, Matsunami Glass Ind.). Incisors were held between two pipette tips in the medium. The images were obtained by fluorescence microscopy (BZ9000, Keyence Corp.) using 10× objective every 10 min for 12 h at 37 °C in a humidified atmosphere of 5% CO_2_ within a stage top incubator (Tokai Hit). The time-lapse images were processed using Aivia software (Leica Microsystems). Cells were identified using pixel classifier and individual cell tracking was performed with tdTomato-positive cells and Hoechst33342-positive cells respectively, by Aivia. Directional plots were performed and the data were exported to excel for making graphs.

### Statistical analyses

Statistical analyses were performed using GraphPad Prism 9 (GraphPad Software Inc.). To compare two groups, statistical differences were assessed using an unpaired Student’s t-test. To compare three groups, statistical differences were assessed using a one-way ANOVA with Dunnett’s multiple comparisons test. Data are presented as mean ± SD. *p* values < 0.05 were considered statistically significant.

## Results

### Generation of Amelx-promoter tdTomato (AT) mice

**Figure 1:**
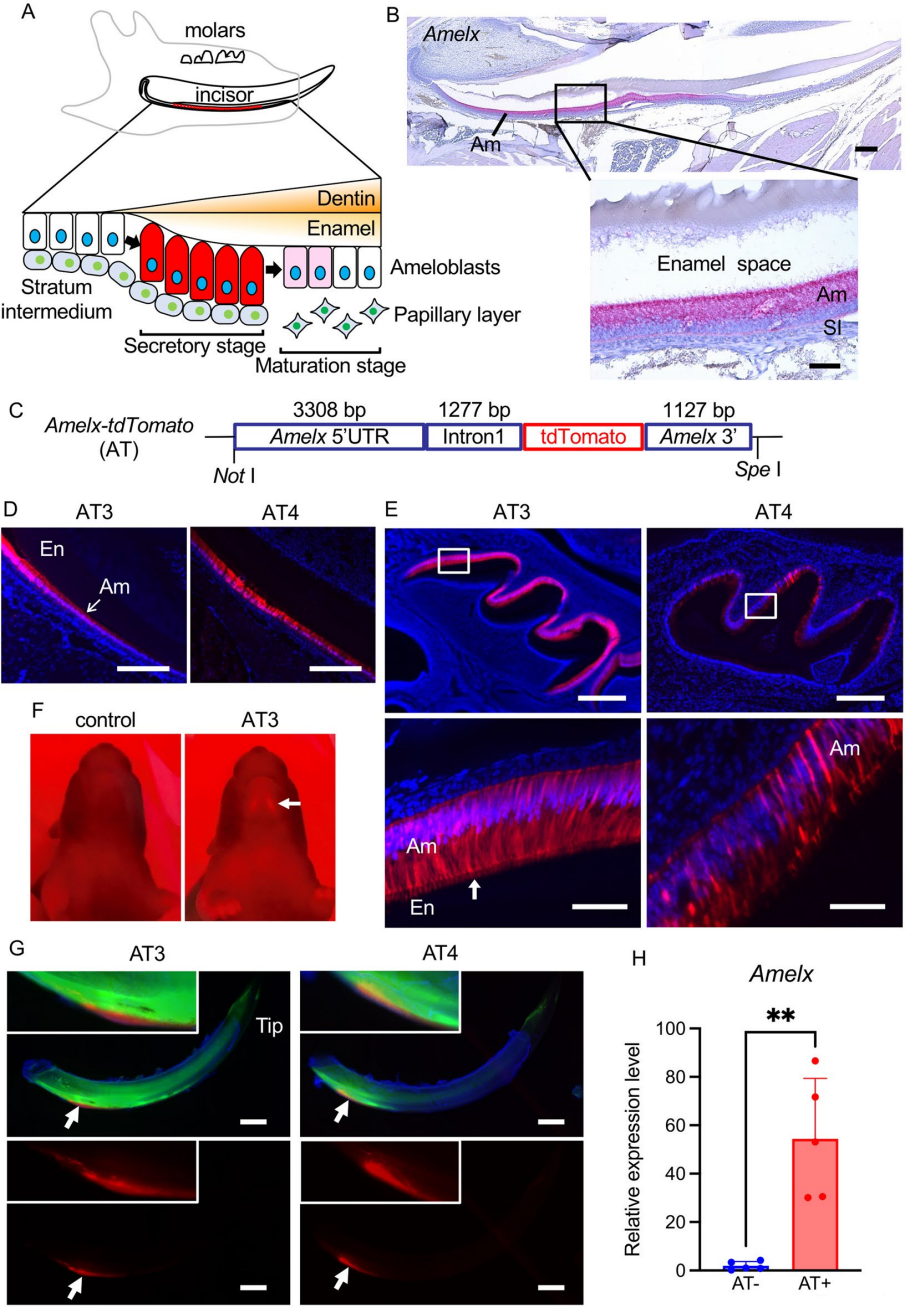
Generation of Amelx-promoter tdTomato (AT) mice. (**A**) An illustration of amelogenesis stages with the secretory stage highlighted in red to represent *Amelx*-promoter driven tdTomato expression. (**B**) *Amelx *in situ hybridization in an adult mouse incisor. Scale bars: 200 μm (upper); 50 μm (lower). (**C**) Schematic of the *Amelx-tdTomato* reporter construct. (**D**) Adult mouse incisor frozen sections showing the high tdTomato expressing transgene (AT3) and the low expressing transgene (AT4); blue, DAPI stain. Scale bars: 300 μm. (**E**) Mouse mandible frozen sections at the age of five days showing the AT3 and AT4; blue, DAPI stain. Boxes inside panels are magnified in panels beneath. A vertical arrow indicates Tomes’ processes. Scale bars: 300 μm (upper); 50 μm (lower). (**F**) Photos of control and AT3 5 day old mouse mandibular area illuminated by an LED light (LED530-3WRF, OptoCode). A horizontal arrow indicates the region where tdTomato is expressed. (**G**) Mandibular incisors from 7-week-old mice that were calcein labeled by intraperitoneal injections (10 mg/kg; Dojindo) and dissected. The incisors were stained with Hoechst33342 and observed using fluorescence microscopy without fixation. Upper panels were merged with tdTomato (red), calcein (green) and Hoechst33342 (blue). Lower panels are labeled with tdTomato. Arrows indicate regions of tdTomato expression and the regions were magnified in the boxes located to the left top side. Scale bars: 1 mm. (**H**) Relative gene expression level of amelogenin was analyzed by qPCR to compare between the sorted AT ( +) and AT (−) cells (n = 5 mice per group). *Gapdh* was used to normalize the results. Am, ameloblast; SI, stratum intermedium; En, enamel. **p < 0.01.

A schematic illustration of amelogenesis stages is presented with the secretory stage highlighted in red to represent *Amelx*-promoter driven tdTomato expression (Fig. 1A). We confirmed by in situ hybridization that *Amelx* mRNA distribution was restricted to ameloblasts in mouse incisor (Fig. 1B) and this was previously demonstrated in molar ameloblasts^20^. To label ameloblasts in vivo, we generated mice transgenic for *Amelx*-promoter driven tdTomato (Fig. 1C). Two transgenic lines, *Amelx-tdTomato*#3 and #4 (AT3 and AT4) were obtained. Both lines specifically expressed tdTomato in adult mouse incisor ameloblasts and first molar ameloblasts at postnatal day 5 (P5) (Fig. 1D, E). Ameloblast Tomes' processes were also tdTomato positive. The tdTomato expression level was high in AT3 ameloblasts compared to AT4. AT3 red fluorescence expression was even visible through the skin of P5 AT3 mice (Fig. 1F). Amelogenesis progressed normally in AT mouse incisors as indicated by normal enamel formation along the continuously erupting incisors (Supplementary Fig. S1). To investigate living ameloblasts by ex vivo imaging, mandibular incisors from AT3 and AT4 mice were carefully dissected from their mandibles (Fig. 1G). The tdTomato positive ameloblasts were on the labial side of the incisors as shown by arrows. The tdTomato started expressing at the same position (arrows in Supplementary Fig. S2) as the calcein labeled regions, which shows that calcification had started. Subsequently, AT4 mice were primarily examined because AT3 mouse tdTomato fluorescence was too bright to assess with single cell resolution. Enamel organ epithelial cells from AT4 mouse incisors were sorted to separate tdTomato-positive and -negative cells (Supplementary Fig. S3). Quantitative PCR (qPCR) analyses demonstrated that genes predominately expressed in ameloblasts were expressed in the tdTomato positive cells, but not in the negative cells. For example, endogenous *Amelx* mRNA expression levels were higher in the tdTomato-positive cells than in the negative cells (Fig. 1H). Similarly, endogenous *Ambn*, and *Mmp20* expression levels were also higher in the tdTomato-positive cells compared to the negative cells (Supplementary Fig. S3). Conversely *Dspp*, which is predominantly expressed in odontoblasts and Cyclin B1 (*Ccnb1*) expression levels (ameloblasts do not replicate) were not statistically different between tdTomato-positive and negative cells (Supplementary Fig. S3). Taken together the *Amelx-tdTomato* mice specifically labeled ameloblasts and therefore may be a useful tool for imaging and isolate mouse ameloblasts.

### Three-dimensional (3D) imaging of mouse ameloblasts

**Figure 2:**
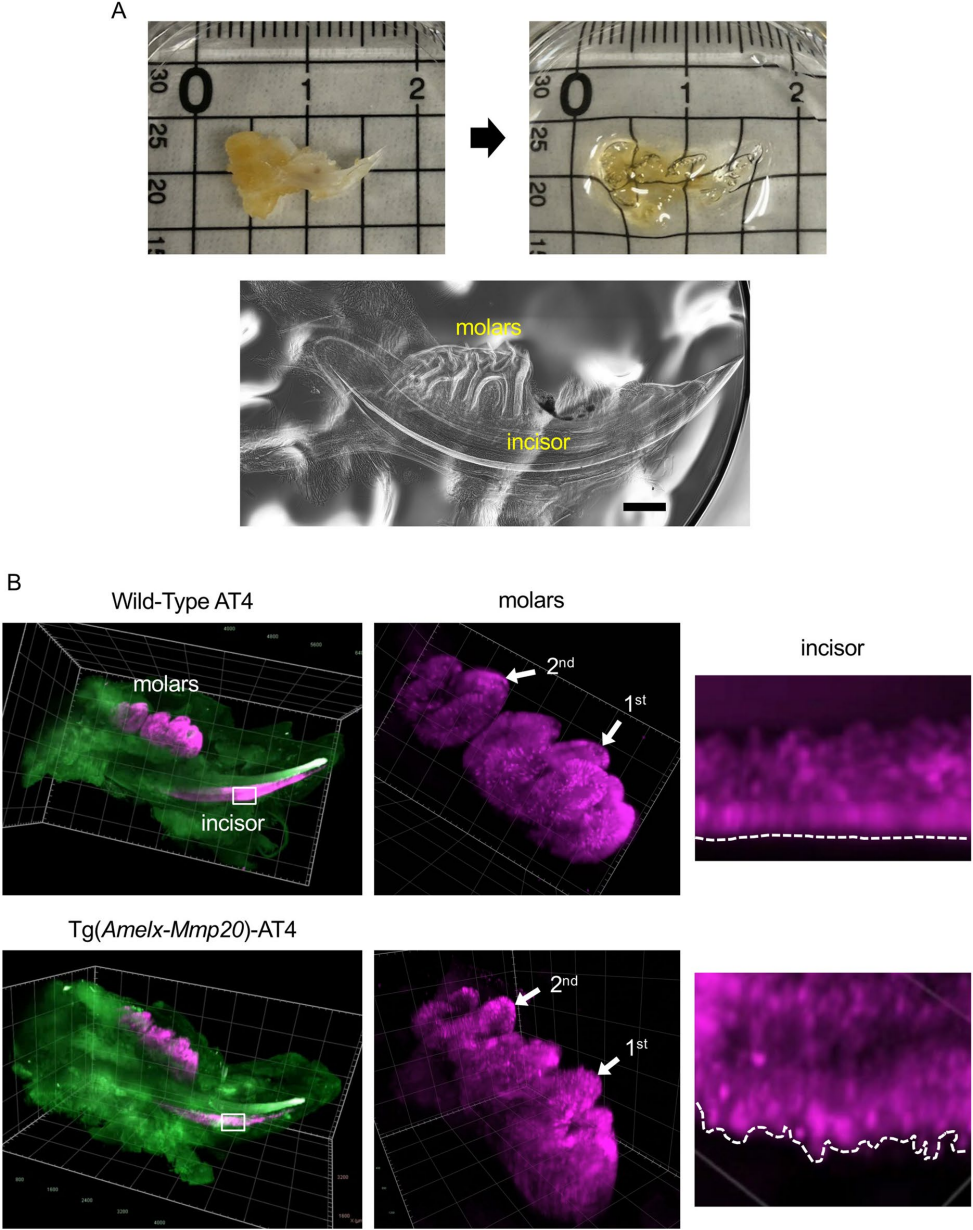
3D imaging of a fixed AT mouse mandible. (**A**) A mandible from a 7-week-old *Mmp20*^+/+^-AT4 mouse was cleared with CUBIC. Upper panels, before (left) and after (right) clearing. Lower panel, phase contrast view of the cleared mandible. Scale bar: 1 mm. (**B**) Cleared mandibles from 5 day old *Mmp20*^+/+^-AT4 and Tg(*Amelx*-*Mmp20*)AT4 mice were imaged using a light-sheet fluorescence microscope. Magenta, tdTomato; green, autofluorescence. The molar and incisor were magnified to better observe the ameloblasts. Dash lines along the incisors outline the buccal surface of ameloblasts.

The AT mice hemi-mandibles were fixed and cleared by CUBIC (Fig. 2A). Molar and incisor morphology were then observed by phase contrast microscopy and the cleared mandibles were observed by standard fluorescence microscopy (Supplementary Fig. S4). tdTomato was expressed in first and second molars at P5 and in third molars at P13. The continuously erupting incisors expressed tdTomato during the secretory stage at all time points examined (P5 through 7 weeks) in AT3 mice. The tdTomato expression in AT4 mice was weaker than that of AT3 mice.

The 3D arrangement of the single cell layer of ameloblasts on AT4 wild-type molars and incisors were observed by light-sheet fluorescence microscopy (Fig. 2B, Supplementary Movies 1, 2, 4, 6). The incisor ameloblasts started expressing tdTomato during early development and the tdTomato-positive area became broader and the fluorescence levels became higher as enamel development progressed further into the secretory stage of enamel formation (Supplementary Movie 6).

Previously we reported that Tg(*Amelx*-*Mmp20*) mice that overexpress MMP20, had a disorganized ameloblast layer^15^. Here we generated Tg(*Amelx*-*Mmp20*)-AT4 mice and examined the ameloblast layer. We confirmed that in three-dimensions the Tg(*Amelx*-*Mmp20*) ameloblast layer arrangement was disorganized as cells had migrated out of the ameloblast layer (Fig. 2B, Supplementary Movie 3, 5, 7). The disorganized ameloblast layer results in producing thin enamel surrounded by disorganized ectopic mineralized nodules^20^.

### Ameloblast gene expression analyses in Mmp20^***−/−***^*** and Tg(Amelx-Mmp20) mice***

**Figure 3:**
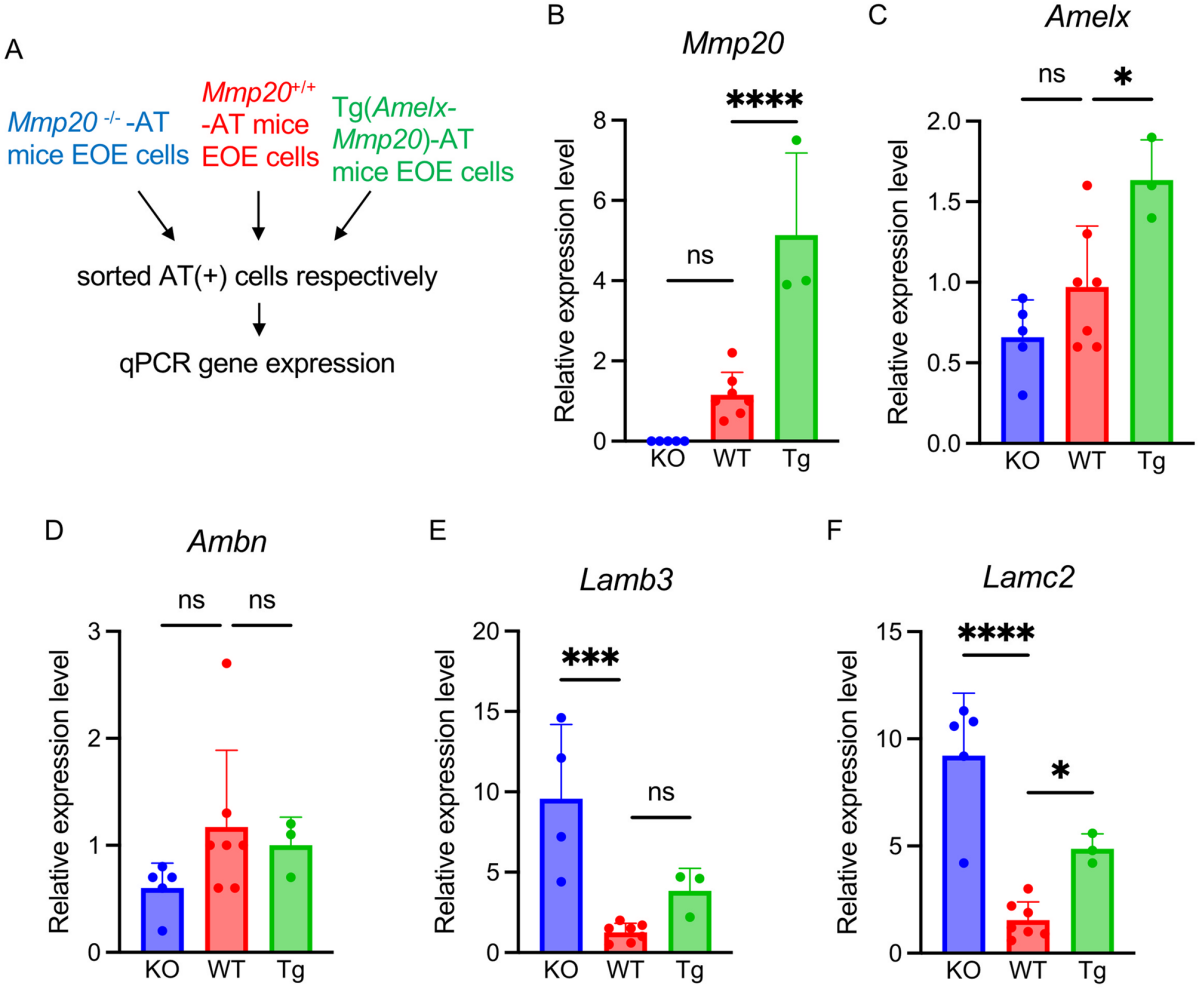
Gene expression analysis with sorted AT positive cells. (**A**) Schematic of AT positive cell isolation and expression analyses. (**B–F**) Relative gene expression levels in ameloblasts isolated from *Mmp20*^*−/−*^-AT (KO), *Mmp20*^+*/*+^-AT (WT), and Tg(*Amelx*-*Mmp20*)-AT (Tg). Cells were analyzed by qPCR. Results are relative to expression levels observed in the *Mmp20*^+*/*+^-AT4 (WT) samples. (*Mmp20*^−/−^-AT4: n = 5 mice, *Mmp20*^+*/*+^-AT4: n = 7 mice, Tg(*Amelx*-*Mmp20*)-AT4: n = 3 mice). Data was normalized to *Gapdh*. ns, not significant; *p < 0.05; ***p < 0.001; ****p < 0.0001.

Here *Mmp20*^−/−^-AT and Tg(*Amelx*-*Mmp20*)-AT mice were generated for gene expression analyses. We isolated tdTomato-positive cells from enamel organ epithelial from the incisors of three mouse lines, which were *Mmp20*^+*/*+^-AT4 (WT), *Mmp20*^−/−^-AT4 (KO), Tg(*Amelx*-*Mmp20*)-AT4 (Tg). From these three genotypes, we quantified gene expression in genes associated with enamel formation, cell migration and cell adhesion by use of qPCR on mRNA extracted from the isolated epithelial cells from each genotype (Fig. 3A).

*Mmp20* expression could not be detected in *Mmp20*^−/−^-AT4 mice, while *Mmp20* expression in Tg(*Amelx*-*Mmp20*)-AT4 mice was approximately five times higher than in *Mmp20*^+*/*+^-AT4 mice (Fig. 3B). *Amelx* expression levels were correlated with *Mmp20* expression levels (Fig. 3C). There was no statistically significant difference in ameloblastin gene (*Ambn*), expression levels between the three mouse lines (Fig. 3D). Gene expression changes during mouse amelogenesis were analyzed by single cell RNA sequence (scRNA-seq)^17^. Cell migration and adhesion genes were selected by Amigo (http://amigo.geneontology.org/amigo) from the scRNA-seq data and analyzed by qPCR (Fig. 3E, F; Table 2). Among those genes, *Lamb3,* encoding Laminin beta 3, was upregulated in *Mmp20*^−/−^ -AT4 and slightly increased in Tg(*Amelx*-*Mmp20*)-AT4 mice (Fig. 3E) compared to expression levels in *Mmp20*^+*/*+^-AT mice (*Mmp20*^−/−^-AT4 = 9.6 ± 4.6, *Mmp20*^+*/*+^-AT4 = 1.3 ± 0.6, Tg(*Amelx*-*Mmp20*)-AT4 = 3.8 ± 1.4). *Lamc2,* encoding Laminin gamma 2, was also upregulated in *Mmp20*^−/−^ -AT4 and Tg(*Amelx*-*Mmp20*)-AT4 mice (Fig. 3F) when compared to expression levels in *Mmp20*^+*/*+^-AT mice (*Mmp20*^−/−^-AT4 = 9.2 ± 2.9, AT4 = 1.5 ± 0.9, Tg(*Amelx*-*Mmp20*)-AT4 = 4.9 ± 0.7). The expression of *Cdh1*, encoding E-Cadherin, was not upregulated during amelogenesis^17^ and the same expression levels were observed in *Mmp20*^−/−^-AT4 and *Mmp20*^+/+^-AT4 incisors. However, *Cdh1* expression was upregulated in Tg(*Amelx*-*Mmp20*)-AT4 (Table 2). *Cdh2*, encoding N-Cadherin, was also expressed at a higher level in Tg(*Amelx*-*Mmp20*)-AT4 incisors compared to *Mmp20*^+/+^-AT4 incisors (Table 2). *Fam83d* (family with sequence similarity 83 member D) expression was also upregulated in Tg(*Amelx*-*Mmp20*)-AT4 (Table 2).

*Cd55* and *Cntnap2* (contactin associated protein 2) were upregulated in Tg(*Amelx*-*Mmp20*)-AT4 incisors (Table 2), while *Runx1* (runt related transcription factor 1) was not statistically different between the three mouse lines (Table 2). Sixteen additional genes associated with cell migration and cell adhesion were compared for expression levels in *Mmp20*^−/−^-AT4 and *Mmp20*^+/+^-AT4 mouse incisors (Table 2). *Mfge8* (milk fat globule EGF and factor V/VIII domain containing), *Mpzl2* (myelin protein zero like 2), *Perp* (p53 apoptosis effector related to PMP22), *Pstpip1* (proline-serine-threonine phosphatase interacting protein 1), *Gja1* (gap junction protein alpha 1), *Pfn1* (profilin 1) and *St14* (suppression of tumorigenicity 14) were expressed at higher levels in *Mmp20*^−/−^-AT4 compared to *Mmp20*^+/+^-AT4 incisors. Conversely, the *S100a10* (S100 calcium binding protein A10), *Tmsb10* (thymosin beta 10) and *Tmsb4x* (thymosin beta 4 X-linked) genes were expressed at lower levels in *Mmp20*^−/−^-AT4 incisors compared to *Mmp20*^+/+^-AT4 incisors. *Rps3* (ribosomal protein S3), *Rps19* (ribosomal protein S19), *Spint2* (serine peptidase inhibitor, Kunitz type 2), *Cd81*, *Sparc* (secreted protein acidic and cysteine rich) and *St3gal4* (ST3 beta-galactoside alpha-2,3-sialyltransferase 4) were not statistically different between *Mmp20*^−/−^-AT4 and *Mmp20*^+/+^-AT4 incisors.

Taken together these gene expression analyses in the ameloblast tdTomato-positive cell population, revealed several genes associated with cell migration and adhesion display altered expression in ameloblasts when *Mmp20* is absent and when *Mmp20* is overexpressed. This suggests that MMP20 plays a significant role in ameloblast cell migration and adhesion.

### Amelx-tdTomato positive cell live imaging

One of the advantages of AT mice is that we can observe live tdTomato-positive ameloblasts by fluorescence microscopy. Here we investigated ameloblast morphology and movement in *Mmp20*^+/+^-AT4 mouse mandibles ex vivo.

**Figure 4:**
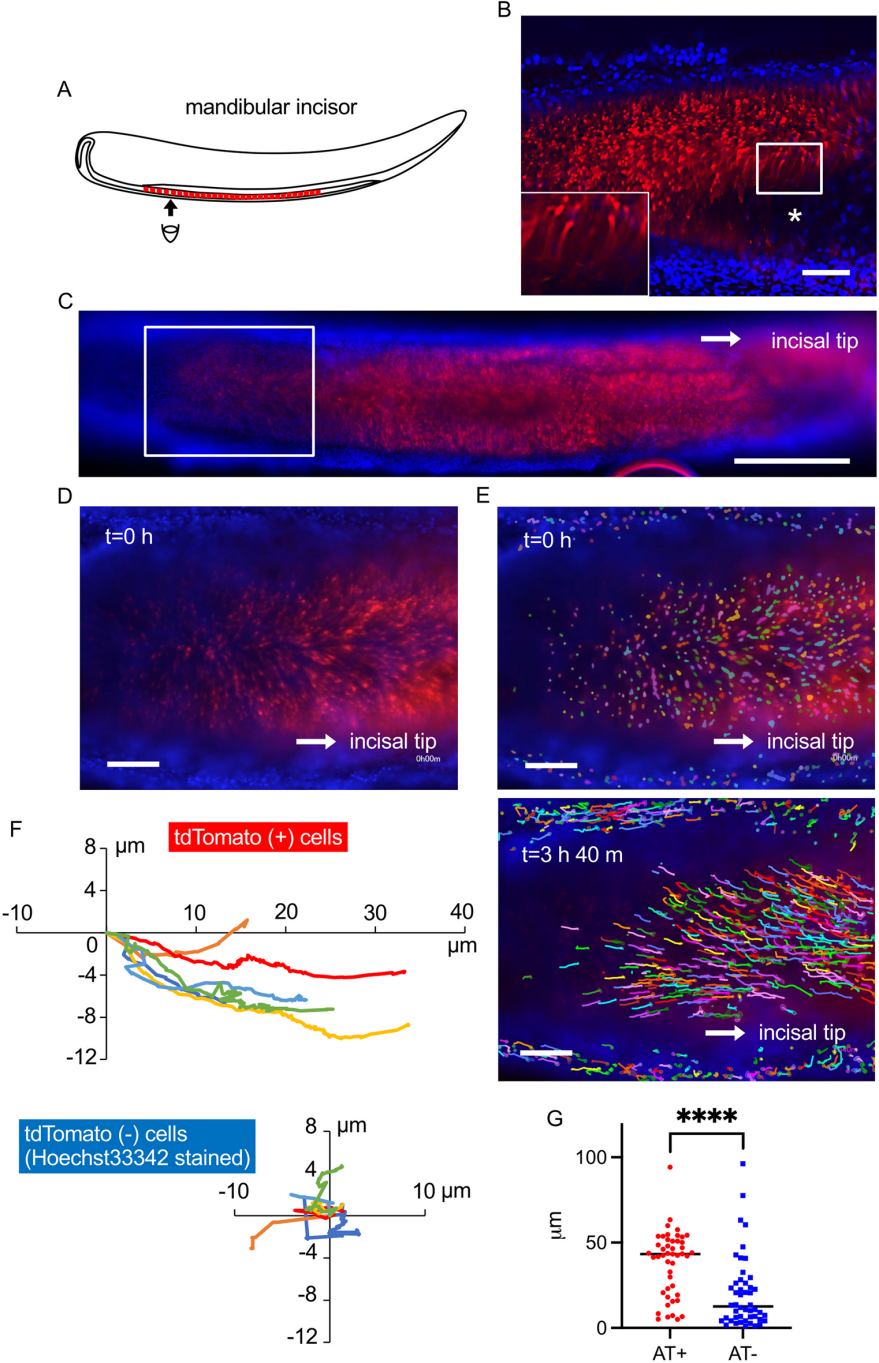
Morphology and movement of mouse incisor ameloblasts. (**A**) An illustration of a longitudinal view of a mandibular incisor. A mandibular incisor was extracted from a 5-day-old *Mmp20*^+*/*+^-AT4 mouse. (**B**) Lateral incisor view. Elongated live tdTomato positive (red) ameloblasts in a small box above the asterisk, was magnified in the larger box in panel (**B**) located to the left bottom side. The extracted incisor was stained with Hoechst33342 (blue) and the surrounding non-ameloblast cells, which are located on the upper and lower sides of the red tdTomato, stained blue. An asterisk shows a crack that occurred during incisor extraction. (**C**) A lower magnification view from the same plane as (**B**). (**D**) Time-lapse imaging of tdTomato positive cells (ameloblasts) and the surrounding cells stained with Hoechst33342. (**E**) Individual tdTomato positive ameloblasts are shown with surrounding cells stained with Hoechst33342. These cells from panel (**D**) were marked with different colors and the cells were tracking for 3 h 40 min by Aivia. (**F**) Movement of the tdTomato positive cells (upper) and the surrounding cells stained with Hoechst33342 (lower) were plotted. Ameloblasts moved toward incisal tip. Conversely, the surrounding non-ameloblast cells stayed close to the same the locations. Six representative cells were shown per group. (**G**) Cell movement distance was assessed for 12 h and was compared between the tdTomato positive cells and the Hoechst33342 stained cells. tdTomato (+) n = 47, tdTomato (−) n = 48. ****p < 0.0001. Scale bars: 100 μm (**B,D,E**); 500 μm (**C**).

The unerupted molars at P5 could not be clearly observed in single cell resolution (Supplementary Fig. S5). Therefore, incisors were extracted from mandibles and were viewed from the buccal side (Fig. 4A). Elongated and sometimes curved individual ameloblasts could be observed when visualized at high magnification (Fig. 4B, Supplementary Movie 8). The mouse incisor has a rounded shape that precludes low magnification visualization. Therefore, images were z-stacked for full focus visualization (Supplementary Fig. S6), and joined to the adjacent images to observe the majority of the tdTomato-positive cells on the incisor (Fig. 4C). At high magnification within a single plane, time-lapse imaging was performed on the incisor ameloblasts (Fig. 4D, E, Supplementary Movies 9, 10). *Mmp20*^+/+^-AT positive cells and surrounding *Mmp20*^+/+^-AT negative cells that are not ameloblasts were stained with Hoechst33342 and were pseudo-colored. *Mmp20*^+/+^-AT positive ameloblasts moved forward toward the incisal tip, while *Mmp20*^+/+^-AT negative cells stayed in place or moved in several directions (Fig. 4F, G). Using the ex vivo imaging system, living ameloblast morphologies and cell movement were successfully visualized.

## Discussion

In this study we have generated reporter mice that use an *Amelx*-promoter driven tdTomato transgene to label mouse ameloblasts. There are previous reports that created reporter mice to label ameloblasts^21–23^. The first generated *Enamelin-tdTomato* transgenic reporter mice^22^. This study focused on strategies to assess ameloblast differentiation. The second study generated *Amelx-tdTomato* knock-in mice that were crossed with *Dspp-GFP* mice to identify ameloblasts and odontoblasts simultaneously^21^. Note that both *Enamelin-tdTomato* and our *Amelx-tdTomato* constructs were expressed in P5 1st molar secretory stage ameloblasts. The third study generated multiple reporter mice with ameloblast-stage specific Cre recombinases^23^. We generated two different *Amelx-tdTomato* transgenic mouse lines with two different tdTomato expression levels (Table 1) and each of our transgenic mice were useful depending on the experimental protocol.

**Table 1:** Comparison between *Amelx-tdTomato*3 (AT3) and *Amelx-tdTomato*4 (AT4).

	*Amelx-tdTomato3*	*Amelx-tdTomato4*
Expression levels	+ +	+
Expression patterns	All ameloblasts	Amounts vary in each ameloblast
Ease of imaging	−	+
As a marker	Marginal	Acceptable

AT3 and AT4 expressed tdTomato specifically in the ameloblasts. Differences in the tdTomato expression levels are probably from differing copy numbers of each transgene.

With our AT mouse system and tissue clearing technique, we were able to observe mouse ameloblast 3D structures by use of Tg(*Amelx*-*Mmp20*)-AT4 mice (Fig. 2, Supplementary Movies 2–7). The live tdTomato-labeled ameloblasts had a curved shape with both their proximal and distal ends inclined (Fig. 4B). These results are consistent with previous observation of ameloblast shape using freeze-fracture methods^24^. Furthermore, we could observe ex vivo ameloblast movement in *Mmp20*^+/+^ -AT4 mouse incisors by time-lapse imaging (Fig. 4D–F, Supplementary Movies 9, 10). It is thought that epithelial cells undergo a partial epithelial-to-mesenchymal transition (EMT) prior to moving as a sheet and then use guidance signals to facilitate cell migration^25^. Ameloblasts move back to allow appositional growth of the forming enamel layer. Ameloblasts also move to establish the enamel shape and, in the case of mouse incisors, ameloblasts move toward the incisal tip during the secretory stage of enamel development^8^. In this study pre-secretory stage ameloblasts, which started expressing *Amelx-tdTomato* could be observed to migrate as they entered the secretory stage (Fig. 4D–F, Supplementary Movies 9, 10). The incisor ameloblast movement speed, as assessed by time-lapse imaging, was quicker than incisor growth, which is approximately 0.4 mm per week in one week old mice^17^. Ameloblasts move back for appositional growth of the enamel layer, move forward with the incisor, and move in directional cohorts that slide by one another to form the decussating enamel prism pattern. This is likely why they move faster than the erupting incisor.

Gene expression changes during amelogenesis were recently examined by single cell RNA-seq^17^. From the gene expression data set, we chose cell adhesion and migration related genes to analyze because these genes are candidates that may regulate ameloblast layer arrangement and cell migration. We used *Mmp20*^+/+^-AT4, *Mmp20*^−/−^-AT4 and Tg(*Amelx*-*Mmp20*)-overexpressing AT4 ameloblasts to compare gene expression levels by qPCR (Fig. 3, Table 2).

**Table 2:**
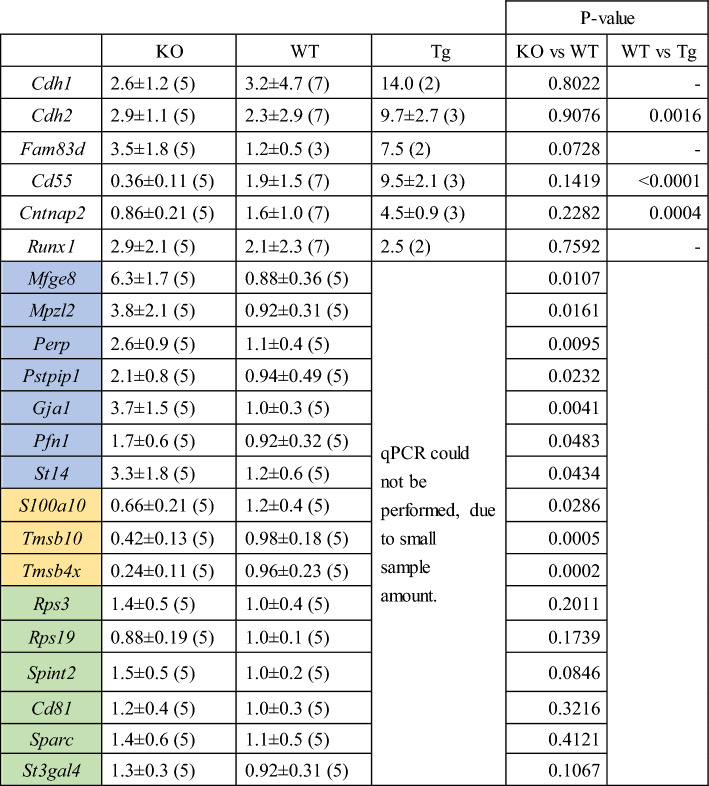
Expression levels of genes involved in cell migration and adhesion in *Mmp20*^−/−^-AT + (KO), *Mmp20*^+*/*+^-AT + (WT) and Tg(*Amelx-Mmp20*)-AT + (Tg) cells.

Relative gene expression levels were analyzed by qPCR relative to expression levels in the *Mmp20*^+*/*+^-AT4 (WT) mouse samples. Results were normalized to *Gapdh* expression. The numbers of total RNA samples are shown in parentheses. Genes highlighted in blue are upregulated in KO; yellow are downregulated in KO and green show no expression level differences in KO compared to WT.

*Mmp20*^−/−^ mice have malformed enamel rod patterns suggesting that the ameloblasts do not migrate properly^13^. Here we show that in ameloblasts isolated from Tg(*Amelx*-*Mmp20*)-AT4 mice, *Cdh1* and *Cdh2* mRNA expression levels are up-regulated compared to AT4 positive ameloblasts isolated from wild-type mice (Table 2). Perhaps this is because MMP20 cleaves these cadherins^14^ and their expression is upregulated to compensate for excess cleavage by the MMP20 overexpressing Tg mice. Mutations in either *LAMB3* or *LAMC2* cause enamel malformation, and *Lamb3* and *Lamc2* encode components of Laminin332 that are required for ameloblast attachment to extracellular matrix^26^. Both of these laminin332 components were upregulated in ameloblasts isolated from MMP20^−/−^ mice, which may indicate the presence of a feedback mechanism in an attempt to overexpress essential enamel formation components to compensate for the lack of MMP20.

Our data in this study suggests that MMP20 may be responsible for turnover of Laminin proteins. Here we assessed mRNA expression levels and not protein cleavage activity. So further analysis is needed to demonstrate that MMP20 cleaves Laminin proteins. However, our data suggests that MMP20 may play a role in regulating cell movement and adhesion through a feedback mechanism during amelogenesis.

Our gene expression analyses in the cell sorted tdTomato-positive cell population (ameloblasts) demonstrated that MMP20 changes several gene’s expression patterns that are associated with cell migration and adhesion, which suggests that MMP20 plays a significant role in ameloblast cell migration and adhesion. Additionally, several genes were identified that were not previously reported as functioning during amelogenesis (*Cd55*, *Cntnap2*, *Mfge8*, *Mpzl2*, *Pstpip1*, *Pfn1*, and *S100a10*).

Two limitations in this study are that *Amelx* expression levels were different between the *Mmp20*^*−/−*^ mice and the Tg(*Amelx*-*Mmp20*) over expresser mice (Fig. 3C). This could occur from an MMP20 feedback mechanism that regulates *Amelx* expression. However, it is also possible that the tdTomato-positive cells sorted from the different genotypes may not have come from exactly the same developmental stage, which may have affected the qPCR gene expression results. Future experiments will need to confirm the observed gene expression changes associated with mouse genotype. Second, we are seeking ways to improve the ameloblast incisor time-lapse results to better define migration differences among *Mmp20* genotypes.

In summary, live cell imaging by using two-photon fluorescence microscopy on AT positive mouse ameloblasts can be used to elucidate ameloblast movement during amelogenesis. Our *Amelx*-promoter driven tdTomato transgenic mice enables observation of ameloblasts in molars and incisors from different planes and enables high resolution time-lapse 3D cell imaging of secretory stage ameloblast movement. These mice also enable isolation of ameloblasts from enamel organ-derived cell populations. Additionally, novel genes functioning during amelogenesis were discovered in this study that may encode critical molecules necessary for ameloblast movement and/or amelogenesis. Future studies using these mice may facilitate the discovery of novel molecular mechanisms that promote amelogenesis through cell tracking techniques or through isolation of a pure population of ameloblasts for molecular mechanistic studies.

### Supplementary Information

Supplementary Information.

Supplementary Video 1.

Supplementary Video 2.

Supplementary Video 3.

Supplementary Video 4.

Supplementary Video 5.

Supplementary Video 6.

Supplementary Video 7.

Supplementary Video 8.

Supplementary Video 9.

Supplementary Video 10.
