# Supplementary material for: Mobility gene expression differences among wild-type, Mmp20 null and Mmp20 over-expresser mice plus visualization of 3D mouse ameloblast directional movement
Source: Sci Rep. 2023 Nov 1;13:18829. doi: 10.1038/s41598-023-44627-0 (PMC10620228; doi:10.1038/s41598-023-44627-0)
Supplement: Supplementary file 1 — Supplementary Information. [file 41598_2023_44627_MOESM1_ESM.docx]

**Mobility Gene expression differences among wild-type, *Mmp20* null and *Mmp20* over-expresser mice plus visualization of 3D mouse ameloblast directional movement**

Masashi Shin, Aya Matsushima, Jun-ichi Nagao, Yoshihiko Tanaka, Hidemitsu Harada, Koji Okabe & John D. Bartlett

**Supplementary Information**

**Supplementary Materials and Methods**

**Micro-computed Tomography (μCT)**

Mouse hemi-mandibles were fixed in 4% paraformaldehyde and scanned using μCT (SkyScan 1176; Bruker BioSpin). Reconstruction was performed using the NRecon software (Bruker microCT). Following reconstruction, 3D image processing and analysis was performed using DataViewer and the CTAn software (Bruker microCT).

**Supplementary movie 1.** **Mandibular incisors from *Mmp20^+/+^* -AT4 mice at P5 were cleared by immersion in CUBIC reagents and were observed by light-sheet fluorescence microscopy.** Slides from hemi-mandibles were viewed consecutively in the movie. Ameloblasts in incisor and 1^st^ and 2^nd^ molars labeled with tdTomato (magenta) were observed with single cell resolution. Green, auto fluorescence.

**Supplementary movie 2.** **3D image of cleared *Mmp20^+/+^* -AT4 mouse mandible (same sample as Supplementary movie 1).** The whole hemi-mandible 3D images were constructed and rotated. Magenta, tdTomato and green, auto fluorescence.

**Supplementary movie 3.** **3D image of cleared Tg(*Amelx-Mmp20*)-AT4 mouse mandible.** The whole hemi-mandible 3D images were constructed and rotated. Magenta, tdTomato and green, auto fluorescence.

**Supplementary movie 4.** **3D image of *Mmp20^+/+^* -AT4 mouse 1st and 2nd molars.** Higher magnification of Supplementary movie 2.

**Supplementary movie 5.** **3D image of Tg(*Amelx-Mmp20*)-AT4 mouse 1^st^ and 2^nd^ molars.** Higher magnification of Supplementary movie 3. The 3D ameloblast layer arrangement is abnormal in the Tg*(Amelx-Mmp20)* mouse molars.

**Supplementary movie 6.** **3D image of *Mmp20^+/+^* -AT4 mouse incisors.** Higher magnification of Supplementary movie 2.

**Supplementary movie 7.** **3D image of Tg(*Amelx-Mmp20*)-AT4 mouse incisors.** Higher magnification of Supplementary movie 3. The 3D ameloblast layer arrangement is abnormal in the Tg(*Amelx-Mmp20*) mouse incisor.

**Supplementary movie 8.** **Z-stack image of living ameloblasts in *Mmp20^+/+^* -AT4 mouse incisors.** The extracted incisor is viewed from the buccal side. Each red dot is a tdTomato-positive ameloblast. Ameloblasts are seen as elongated and curved. The cells surrounding the ameloblasts were stained with Hoechst33342 (blue).

**Supplementary movie 9.** **Time-lapse imaging of ameloblasts in *Mmp20^+/+^* -AT4 mouse incisors.** The extracted incisor was viewed from the buccal side. Images were taken at 10 min intervals for 12 h. Red, tdTomato and blue, Hoechst33342.

**Supplementary movie 10.** Each cell from Supplementary movie 9 was pseudo-colored. Both tdTomato-positive ameloblasts and the surrounding Hoechst33342 stained cells were pseudo-colored and the cell tracks were visualized. AT positive ameloblasts moved forward toward the incisal tip (right side) whereas the Hoechst33342 stained cells did not.

**Supplementary Figure S1. μCT of adult AT mouse incisors.** (A) The hemi-mandibular incisors were reconstructed. Left, sagittal plane of the hemi-mandibles. Right, the coronal plane at locations indicated by dashed lines in the left panels. De, dentin; En, enamel. (B) Enamel thickness from right panels was measured. Mandibles were analyzed from 3 mice per genotype. There were no statistically significant differences between control and AT mice. (C) Enamel mineral density was measured where the incisors erupt from the mandibular bone . Enamel density was measured from 3 controls, 3 AT4 and 2 AT3 mice. No statistically significant difference was present between control and AT4 mice.

**Supplementary Figure S2. *Mmp20^+/+^* -AT3 mouse incisors at postnatal day 5 (P5, upper panels) and day 13 (P13, lower panels).** *Mmp20^+/+^* -AT3 mice were calcein labeled by intraperitoneal injections. The mandibular incisors were dissected from *Mmp20^+/+^* -AT3 mice and stained with Hoechst33342. Red, tdTomato; green, calcein and blue, Hoechst33342. Arrows indicate calcein label starting points.

**Supplementary Figure S3. The tdTomato construct allows the effective isolation of ameloblasts from enamel organ epithelial cells.**

Isolation of *Mmp20^+/+^* -AT(+) cells and *Mmp20^+/+^* -AT(-) cells. (A) The tdTomato positive and negative enamel organ epithelium cells from *Mmp20^-/-^* -AT4, *Mmp20^+/+^* -AT4 and Tg(*Amelx-Mmp20*)-AT4 mice were sorted by FACSMelody. (B) Gene expression levels were compared between *Mmp20^+/+^* -AT(+) and *Mmp20^+/+^* -AT(-) cells by qPCR (n=5 mice). *Gapdh* was used for normalization. **p<0.01. P values are shown in the graph to the right. *Ambn* is expressed from ameloblasts and *Dspp* is expressed from odontoblasts.

**Supplementary Figure S4. Cleared *Mmp20^+/+^* -AT mouse mandibles at P5, P13 and 7 weeks old.** Fixed *Mmp20^+/+^* -AT3 and *Mmp20^+/+^* -AT4 mouse mandibles were cleared and observed by fluorescence microscopy at indicated time points. Arrows at the 7 week old time point indicate tdTomato positive secretory stage ameloblasts at the labial side of the incisors.

**Supplementary Figure S5. Live imaging of *Mmp20^+/+^* -AT4 unerupted molars in P5 mandibles.** Occlusal view of *Mmp20^+/+^* -AT4 mouse mandible. Upper left window is a higher magnification of the boxed area. Ameloblasts are recognized as single dots in the high magnification regions. However, it was difficult to recognize single cells in the low magnification regions. Red, tdTomato.

**Supplementary Figure S6. Buccal view images of an *Mmp20^+/+^* -AT mouse P6 incisor.** Extracted *Mmp20^+/+^* -AT4 mouse incisor images were taken at 20 different depths, overlaid and full-focused. Red, tdTomato; blue, Hoechst33342.

**Supplementary Table S1. Primer sets used for qPCR.**

| Gene | 5'-forward-primer-3' | 5'-reverse-primer-3' |
| --- | --- | --- |
| *Amelx* | AGCATCCCTGAGCTTCAGACAGA | AACCAGGGCTTCCAGGATGAG |
| *Mmp20* | CCTCCCCAAGGATGATGTGAAAGG | TCACTGCATCAAAGGACGAGCTGG |
| *Ambn* | TTCCAGATCGCCCGTTCAA | ATGAAGCCAATTCTGGCAGGAG |
| *Lamb3* | GAGGGGCCTGTTATCCACCT | CTGCCACTGTCCATATTGGGT |
| *Lamc2* | CGAGACCAGGGGCAACTAGA | GGTCGGCACCTATCACAGC |
| *tdTomato* | AGCAAGGGCGAGGAGGTCAT | CCTTGGAGCCGTACATGAACTGG |
| *Dspp* | ATTCCGGTTCCCCAGTTAGTA | GGGAGTCGTTGCTGTTGCTA |
| *Ccnb1* | GCGTGTGCCTGTGACAGTTA | CCTAGCGTTTTTGCTTCCCTT |
| *Cdh1* | CAGTTCCGAGGTCTACACCTT | TGAATCGGGAGTCTTCCGAAAA |
| *Cdh2* | AGGCTTCTGGTGAAATTGCAT | GTCCACCTTGAAATCTGCTGG |
| *Fam83d* | ATGACAGTTCGGACAATTACAGG | CGGATTCCGTCAATCAGTGTGA |
| *Cd55* | ACCCCGGTGCATAGAGAAATC | GGATGACGTACTGTTGTCTTGG |
| *Cntnap2* | CCTTGGCACCTAGATCACTTG | GCGATGACCCCTCCAATGATA |
| *Runx1* | GCAGGCAACGATGAAAACTACT | GCAACTTGTGGCGGATTTGTA |
| *Mfge8* | CCGCGTCTGGTGACTTCTG | TCCTCTCTCAGTCTCATTGCAC |
| *Mpzl2* | AGCCCTTTGTCCTACAGAAGC | GTCACAGTTAGCGCATCTCC |
| *Perp* | ATCGCCTTCGACATCATCGC | CCCCATGCGTACTCCATGAG |
| *Pstpip1* | TGGGCTGCTACATGGAAGTC | TGCGTAGACCAACTCATTCCG |
| *Gja1* | CTGAGTGCGGTCTACACCTG | GAGCGAGAGACACCAAGGAC |
| *Pfn1* | TGGAACGCCTACATCGACAG | GTAATGCTAACGAAGGTCTTCCC |
| *St14* | CTGGATGCGTATGAGAACTCC | TACAGCCGACTTCTTGTGGTA |
| *Rps3* | ATGGCGGTGCAGATTTCCAA | GTAACTCGGACTTCAACTCCAG |
| *Rps19* | GGTTCTACACACGAGCTGCTT | CACACTCTTAGAGCCTCTGCT |
| *Spint2* | TCCCTCGCTGGTACTATGACA | GGGAGAGGTAACTGTTCTTGTTG |
| *Cd81* | GCTCTTCGTCTTCAATTTCGTCT | TGTTGGGTGCCGGTTTGTT |
| *Sparc* | GTGGAAATGGGAGAATTTGAGGA | CTCACACACCTTGCCATGTTT |
| *St3gal4* | ACCAGCAAATCTCACTGGAAG | CCCTGGAAGCATGGCTCTTTC |
| *S100a10* | GCTTACGTTTCACAGGTTTGC | AAGCCCACTTTGCCATCTCG |
| *Tmsb10* | CCGGACATGGGGGAAATCG | CCTGTTCAATGGTCTCTTTGGTC |
| *Tmsb4x* | ATGTCTGACAAACCCGATATGGC | CCAGCTTGCTTCTCTTGTTCA |
| *Gapdh* | TGTGTCCGTCGTGGATCTGA | TTGCTGTTGAAGTCGCAGGAG |
